# Quantitative imaging biomarkers for dural sinus patterns in idiopathic intracranial hypertension

**DOI:** 10.1002/brb3.613

**Published:** 2017-01-03

**Authors:** Dinah Zur, Reut Anconina, Anat Kesler, Svetlana Lublinsky, Ronen Toledano, Ilan Shelef

**Affiliations:** ^1^ Division of OphthalmologySackler Faculty of MedicineTel Aviv Sourasky Medical CenterTel Aviv UniversityTel AvivIsrael; ^2^ Diagnostic Imaging DepartmentSoroka University Medical CenterBen‐Gurion University of the NegevBeer‐ShevaIsrael; ^3^ Zolotowsky Neuroscience CenterBen‐Gurion University of the NegevBeer‐ShevaIsrael; ^4^ Clinical Research CenterSoroka University Medical CenterBen‐Gurion University of the NegevBeer‐ShevaIsrael

**Keywords:** idiopathic intracranial hypertension, MRI, neuro‐ophthalmology

## Abstract

**Objective:**

To quantitatively characterize transverse dural sinuses (TS) on magnetic resonance venography (MRV) in patients with idiopathic intracranial hypertension (IIH), compared to healthy controls, using a computer assisted detection (CAD) method.

**Materials and Methods:**

We retrospectively analyzed MRV studies of 38 IIH patients and 30 controls, matched by age and gender. Data analysis was performed using a specially developed Matlab algorithm for vessel cross‐sectional analysis. The cross‐sectional area and shape measurements were evaluated in patients and controls.

**Results:**

Mean, minimal, and maximal cross‐sectional areas as well as volumetric parameters of the right and left transverse sinuses were significantly smaller in IIH patients than in controls (*p *< .005 for all). Idiopathic intracranial hypertension patients showed a narrowed segment in both TS, clustering near the junction with the sigmoid sinus. In 36% (right TS) and 43% (left TS), the stenosis extended to >50% of the entire length of the TS, i.e. the TS was hypoplastic. Narrower vessels tended to have a more triangular shape than did wider vessels.

**Conclusion:**

Using CAD we precisely quantified TS stenosis and its severity in IIH patients by cross‐sectional and volumetric analysis. This method can be used as an exact tool for investigating mechanisms of IIH development and response to treatment.

## 1 Introduction

Idiopathic intracranial hypertension (IIH) is a disorder of unknown etiology affecting predominantly obese women of childbearing age (Ahlskog, 1982). The diagnosis is established according to the modified Dandy criteria (Smith, 1985). According to proposed diagnostic criteria (Friedman, Liu, & Digre, 2013), IIH can be suspected in the absence of papilledema and abducens nerve palsy, if at least three of four neuroimaging criteria are satisfied. A recent review article calculated pooled sensitivity and specificity for each of these criteria (Bidot et al., 2015): empty sella (80% and 83%, respectively); flattening of the posterior aspect of the globe (66% and 98%); distention of the perioptic subarachnoid space, with (58% and 89%) or without a tortuous optic nerve (43% and 90%); and transverse venous sinus stenosis, which achieved the greatest values of sensitivity and specificity: 97% and 93% respectively.

The etiology of IIH is unknown. The inclusion of transverse venous sinus stenosis as a diagnostic consideration for IIH follows recent cumulating radiologic evidence of cross‐sectional changes in venous outflow. While one study reported an overall incidence of only 20% of venous outflow abnormalities in IIH patients (Johnston, Kollar, Dunkley, Assaad, & Parker, 2002), most publications support the theory of significant outflow obstruction, based on imaging findings, in the majority of IIH cases (Bono et al., 2005; Dwyer, Prelog, & Owler, 2013; Farb et al., 2003; Horev et al., 2013; Rohr et al., 2012). Persistent transverse sinus (TS) stenosis was reported after normalization of ICP, demonstrating a lack of relationship between the caliber of TS and ICP (Bono et al., 2005). In another study, a narrowed TS was shown in thirteen IIH patients with an increase in diameter of all cerebral sinuses after lumbar puncture (Horev et al., 2013). Elsewhere, even compression of the entire dural sinus tree was demonstrated, in addition to TS stenosis, in 88% of 15 patients, which normalized in 47% of them after IIH treatment (Rohr et al., 2012). Dominant‐sided venous obstruction on MRV was described in the majority of pediatric IIH patients (Dwyer et al., 2013).

Quantitative imaging biomarkers (QIB) present reproducible imaging measures that have the ability to detect anatomic changes with high sensitivity, specificity, and accuracy (Prescott, 2013; Smith, Sorensen, & Thrall, 2003). The use of computer‐assisted detection (CAD) in QIB has been shown to be useful for tumor screening, identification of tumor progression (Destounis et al., 2004; Freer & Ulissey, 2001; Macmahon et al., 1999; Patriarche & Erickson, 2007), and for diagnosing and assessing disease progression in Alzheimer disease (Duchesne et al., 2008; Freeborough & Fox, 1998), osteoarthritis (Folkesson, Dam, Olsen, Pettersen, & Christiansen, 2007; Williams et al., 2010), and coronary atherosclerosis (Lin et al., 2012).

To our best knowledge, a CAD scheme for the analysis of dural sinuses on MR venography (MRV) in IIH patients has not been applied systematically. The purpose of this study was to quantitatively characterize transverse dural sinuses in IIH patients, compared to healthy controls, using CAD, and thus attain insight into the pathogenesis and diagnosis of IIH.

## 2 Materials and Methods

This was a retrospective study approved by the local institutional ethics committee, performed at the Tel Aviv Medical Center and the Soroka University Medical Center.

### 2.1 Participants

The database of IIH patients at the Neuro‐Ophthalmology Unit at the Tel Aviv Medical Center was reviewed for adult IIH patients, diagnosed according to modified Dandy criteria (Smith, 1985), who performed MRV between years 2006–2013. Date and opening pressure of lumbar puncture, and treatment at the time of MRV performance were retrieved from patients’ files.

Data of 30 age and gender matched individuals were retrieved from the Radiology Institute at the Soroka University Medical Center. Inclusion criteria for this control group were:

- Normal brain MRI and MRV, determined by a senior neuroradiologist
- No history of brain surgery or sinus vein thrombosis
- No lumbar puncture during the month preceding the imaging

### 2.2 MRI sequences and analysis

#### 2.2.1 Data acquisition

For all subjects MRI was performed with a contrast‐enhanced 3D spoiled; T1w gradient echo sequence. The imaging parameters were TR/TE 5.7/1.75 ms. slice width = 2 mm (reconstructed to 1 mm), in‐plane resolution 0.74 × 1.05 mm, tip angle 30–40 degrees. SENSE reduction factor was applied in most of the studies.

#### 2.2.2 Data analysis

The data were analyzed by a specially developed Matlab algorithm that has been previously described and can be found in the supplemental data (Lublinsky, Friedman, Kesler, Zur, Anconina & Shelef, 2016). Additional manual evaluation of the TS in IIH patients was done using the score developed by Farb et al. (2003). The highest degree of stenosis was evaluated and given a corresponding number from 0 to 4: 0 = discontinuity (gap) or aplastic segment; 1 =  hypoplasia or severe stenosis (<25% of cross‐sectional diameter of the lumen); 2 =  moderate stenosis (25–50%), 3 =  mild stenosis (50–75%); 4 =  no significant narrowing seen (75–100%). A vessel in which stenosis extended to more than 50% of its entire length was defined as a hypoplastic vessel.

### 2.3 Statistical methods

All statistics were computed with SPSS statistical package version 15.0. An independent sample *t*‐test was run that compared cross‐sectional and volumetric data of the study and control groups. A *t*‐test or Mann–Whitney test was run to compare treated and nontreated patients. Pearson correlations were used to evaluate the relationship of cross‐sectional and volumetric data with BMI and age. We used Stepwise Logistic Regression to predict the chance of belonging to the patient group according to cross‐ sectional and volumetric results. ANOVA and Tukey procedure were applied to compare results of manual and computerized analyses.

Statistical analysis was performed by the Statistical Laboratory School of Mathematics, Tel Aviv University, Tel Aviv, Israel.

## 3 Results

### 3.1 Study Population

Forty IIH patients were identified. During image processing, two patients were excluded due to low contrast of the blood vessels. The remaining patients were 34 females and 4 males (See supplemental data for demographic data and baseline characteristics). Mean age was 33 ± 10 (standard deviation, SD) years, mean BMI 32 ± 8 SD. For six patients BMI was not available. The mean opening pressure on lumbar puncture (LP) was 323 mm H_2_O ± 82 SD; in one case (patient number 18), opening pressure was elevated >250 mm H_2_O but the exact measurement was not available. Twenty‐six patients received oral treatment at the time of MRV (Diamox in 22 cases, Topamax in 4 cases), 10 were untreated at the time of MRV, one had a ventriculoperitoneal shunt, and in one case no data were available regarding treatment.

The control group comprised 27 females and 3 males, with normal brain MRI and MRV studies. Mean age was 33 years ± 11 SD. Reasons for referral to MRI‐MRV were suspicion of intracranial vasculitis (*n *= 10), prolonged headaches (*n *= 7), irregularity of intracranial vessels on CTA examination (*n *= 3), trigeminal neuralgia (*n *= 2), paresthesia (*n *= 2), blurred vision (*n *= 2), suspicion of vascular malformation (*n *= 1), eclampsia (*n *= 1), and suspicion of intracranial hypotension (*n *= 1). In one case we were not able to retrieve the reason. In two cases, a LP exam was done 1.5–2 months before MRI‐MRV. In all cases, neurologic exams were normal, as were the optic nerve heads.

### 3.2 MRV analysis

**Figure 1:**
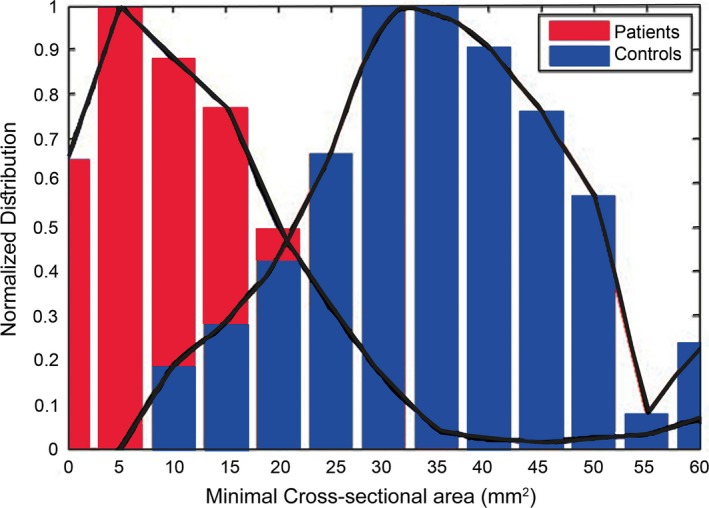
Normalized distribution of minimal cross‐sectional areas. The normalized distribution of minimal cross‐sectional areas, measured along the transverse sinuses for idiopathic intracranial hypertension (IIH) patients and controls

**Figure 2:**
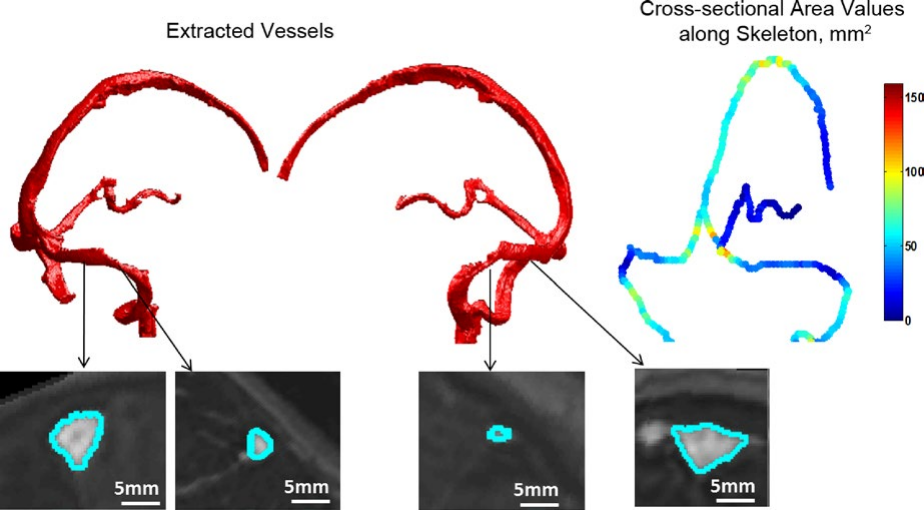
Vessel object in an idiopathic intracranial hypertension (IIH) patient, demonstrating transverse sinus narrowing. Top left and middle: Vessel segmentation and extraction from the brain volume. Two views of the same vessel are shown to demonstrate TS narrowing on both right and left sides. Top right: Distribution of values of cross‐sectional area along skeleton branches. Bottom: Extracted TS cross‐sectional planes are shown

The mean cross‐sectional area of the TS measured 31.6 ± 19.5 SD mm^2^ for patients and 49.7 ± 19.4 SD mm^2^ for controls. The difference between the groups was 38% for the right TS (33.1 mm^2^ in patients vs. 53.3 mm^2^ in controls, *p *< .001) and 35.3% for the left TS (30.3 mm^2^ in patients vs. 46.9 mm^2^ in controls, *p *< .001) (Figure 1). The minimal cross‐sectional areas of the right and left TS in the patients’ group were 67.1% and 74.5% smaller, respectively, than those in the control group (*p *< .001 for both, Figure 2). The maximal cross‐sectional areas were also smaller in patients: by 23.3% (*p *= .002) and 24.6% (*p *= .004) for the right and left TS respectively (Tables 1 and 2).

**Table 1:** Cross‐sectional and volumetric data for the right and left transverse sinuses (RTS and LTS) in patients and controls

	RTS				LTS			
	Patients	Controls	Δ %	*p*	Patients	Controls	Δ %	*p*
Cross‐sectional area								
Mean mm^2^ ± SD	33.1 ± 13.4	53.3 ± 13.3	−37.8 ± 0.8	<.001	30.3 ± 13.5	46.9 ± 12.6	−35.3% ± 7.3%	<.001
Min. mm^2^	11.4	34.7	−67.1	<.001	7.7	30.3	−74.5%	<.001
Max. mm^2^	68.4	89.1	−23.3	.002	66.4	88.1	−24.6%	.004
Minimal area of narrowing								
Mean min. area mm^2^ ± SD	7.8 ± 5.2				7.1 ± 4.3			
Length mm ± SD	53.2 ± 35.9				55.8 ± 36.9			
Start point of narrowed segment as distance from torcula herophili								
Average mm ± SD	28.1 ± 20.8				26.1 ± 21.1			
Volume								
Mean mm^3^ ± SD	3358.5 ± 1469.6	5284.9 ± 1180.0	−36.5 ± 24.6	<.001	3146.0 ± 1204.5	4758.9 ± 1447.3	−33.9 ± 16. 8	<.001
Volume at narrowed region mm^3^								
Mean mm^3^ ± SD	1579.2 ± 855.3				1487.4 ± 680.9			

LTS, Left transverse sinus; RTS, Right transverse sinus; STD, Standard deviation.

**Table 2:** Independent sample *t*‐test comparing cross‐sectional data between IIH patients and controls

	*t*	Sig. (2‐tailed)	95% Confidence interval of the difference	
			Lower	Upper
Right TS mean CS	6.010	<.001	13.4	26.8
	6.108	<.001	13.5	26.7
Right TS minimum CS	8.439	<.001	17.8	28.8
	8.532	<.001	17.9	28.8
Right TS maximum CS	3.252	.002	8.0	33.4
	3.232	.002	7.9	33.5
Left TS mean CS	5.258	<.001	10.4	22.8
	5.127	<.001	10.1	23.0
Left TS minimum CS	10.086	<.001	18.1	27.1
	9.365	<.001	17.7	27.5
Left TS maximum CS	2.994	.004	7.2	36.1
	2.937	.005	6.9	36.5

CS, Cross‐section; EVA ,Equal variances assumed; EVNA, Equal variances not assumed; TS, Transverse sinus; IIH, idiopathic intracranial hypertension.

Idiopathic intracranial hypertension patients exhibited narrowed segments in both TS, clustering near the junction with the sigmoid sinus. Thirty‐three patients (87%) showed a narrowed segment in the right TS, and 37(97%) in the left TS; 85% had a bilateral TS stenosis. Highly significant differences were observed between the patient and control groups in the mean distance of the narrowed segment started from the torcula heterophili, the mean length of the segment, and the mean stenosed length of the transverse sinus (Table 1). The proportion of patients that exhibited a hypoplastic TS was 36% (12/33) for the right TS and 43% (16/37) for the left TS.

The mean cross‐sectional area of the left TS correlated with its triangularity i.e. the narrower the cross‐sectional area, the more triangular its shape (*p *= .013). For the right TS, the correlation did not reach statistical significance (*p *= .079). Among IIH patients with hypoplastic TS (i.e. >50% of the length of the stenosed vessel), mean triangularity scores were 66.5 ± 3.4 SD and 67.9 ± 3.4 SD for the right and left TS, respectively. In IIH patients without hypoplastic TS (i.e. ≤50% of the length of the stenosed vessel) mean triangularity scores were 64.4 ± 2.6 SD and 65.4 ± 4.3 SD for the right and left TS, respectively. TS with a longer stenosis tended to have a more triangular cross‐sectional shape than did TS with a shorter stenosis; the latter tended to have a rounder cross‐sectional shape (right TS: *p *= .05, left TS: *p *= .07).

Transverse sinus volumes were significantly smaller for the patient than the control group (Tables 1 and 3).

**Table 3:** Independent sample *t*‐test comparing volumetric data between IIH patients and controls

	*t*	Sig. (2‐tailed)	95% Confidence interval of the difference	
			Lower	Upper
Right TS volume	5.84	<.001	1268.1	2584.7
	6.00	<.001	1284.8	2567.9
Left TS volume	5.02	<.001	970.9	2255.0
	4.91	<.001	954.6	2271.2
Right + left TS volume	7.15	<.001	2550.3	4528.3
	7.21	<.001	2558.3	4520.3

EVA ,Equal variances assumed; EVNA, Equal variances not assumed; TS, Transverse sinus; IIH, idiopathic intracranial hypertension.

**Figure 3:**
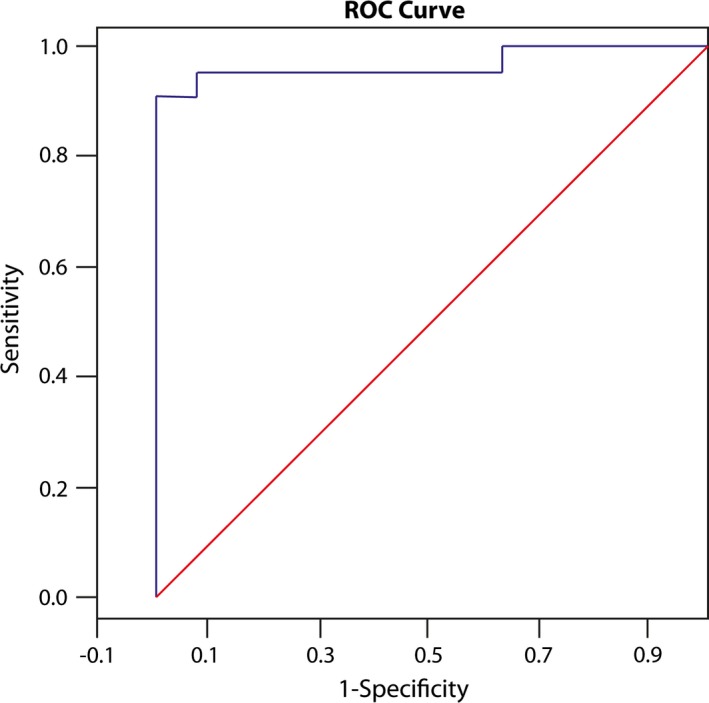
ROC Curve for prediction of belonging to the idiopathic intracranial hypertension (IIH) group. The sensitivity and specificity of the minimal cross‐sectional area of the left TS and the mean cross‐sectional area of the right TS in predicting the chance of belonging to the IIH group (cut‐off value 55%); the area under the ROC curve was 0.98

Using Stepwise Logistic Regression, all cross‐sectional and volumetric parameters showed high prediction of the IIH group. The minimum cross‐sectional area of the left TS and the mean cross‐sectional area of the right TS had the highest predictive value (see Table 4). Assignment to the IIH group was based on a predictive chance >55%. The sensitivity of the test was 97.4%, specificity was 93.3%. Based on these data, the area under the ROC curve was 0.98 (Figure 3).

**Table 4:** Prediction of belonging to idiopathic intracranial hypertension (IIH) group using logistic regression

	*p*	Odds	95% C.I. for odds	
			Lower	Upper
Right TS mean CS area	.025	0.92	0.86	0.99
Left TS mean CS area	<.001	0.75	0.63	0.88

Logistic regression for mean cross sectional area of the right TS and minimal cross‐sectional area of the left TS were highly significant to predict the IIH group.

In the control group, the distribution of cross‐sectional areas measured along the right and the left TS showed two peaks that resulted from the difference between the right and the left transverse sinuses. The LTS was slightly smaller than the RTS, but this difference was not statistically significant. In the patients’ group, the difference between the right and left transverse sinuses was smaller, but still showed a shift of 24 mm between the right and the left TS distribution peaks.

Using Pearson Correlation, we did not find correlations of cross‐sectional and volumetric imaging parameters with patients’ BMI (*p *≥ .100), opening pressure (*p *≥ .095), or age (*p *≥ .077). Imaging parameters did not differ significantly between treated and untreated patients (*p *≥ .124). A subanalysis showed no difference in results between patients who underwent LP less than one month before MRV and those who underwent LP more than one month before MRV.

### 3.3 Comparison of manual and computerized analysis

**Figure 4:**
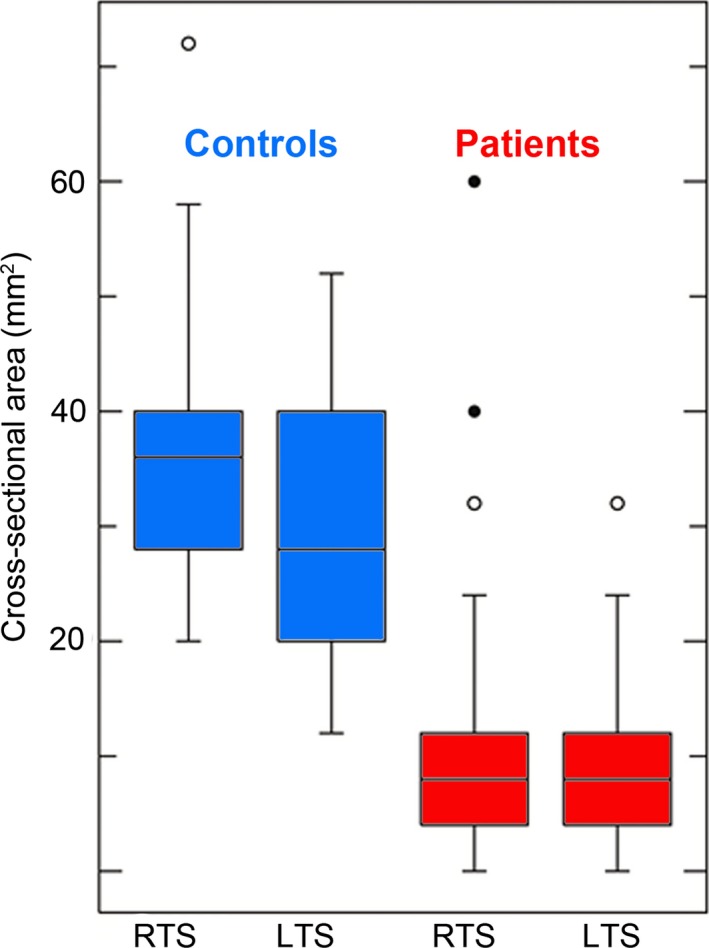
Minimal cross‐sectional area of transverse sinuses in patients and controls. Box plots show the difference in minimal cross‐sectional areas for the right and left TS in patients and controls. RTS‐C, right transverse sinus in controls; LTS‐C, left right transverse sinus in controls; RTS‐P, right transverse sinus in patients; LTS‐P, left transverse sinus in patients

To validate CAD, manual evaluation of the right and left TS was done in 32 IIH patients using a scoring system described by Farb et al. (2003). (Table 5). The correlation between minimal cross sections of the right and left TS with Farb scores reached statistical significance (*p *= .001). Post hoc tests using Tukey's procedure enabled distinguishing between wide and narrow TS cross‐ section areas according to Farb scores (Figure 4).

**Table 5:** Comparison between Farb Score and computer‐assisted detection using ANOVA

	Sum of squares	Mean square	*F*	*p*
Right TS				
Minimum CS area				
Between Groups	953.61	476.80	8.77	.001
Within Groups	1576.27	54.35		
Total	2529.88			
Left TS				
Minimum CS area				
Between Groups	444.42	222.21	9.06	.001
Within Groups	711.45	24.53		
Total	1155.88			

CS, Cross‐section; TS, Transverse sinus.

## 4 Discussion

There is growing evidence that patients suffering from IIH present with cerebral dural sinus stenosis, particularly involving the transverse sinuses. Current data suggest that venous sinus pathology exists in the majority of IIH patients (Bono et al., 2005; Dwyer et al., 2013; Farb et al., 2003; Horev et al., 2013; Rohr et al., 2012). Of all diagnostic imaging criteria, transverse sinus stenosis has been shown to have the highest sensitivity and specificity (Bidot et al., 2015). However, the nature of this sinus narrowing has not been well‐characterized and remains unclear. In this work, we demonstrated significantly narrower transverse sinuses in IIH patients than in controls, based on CAD cross‐sectional and volumetric analyses. Previous reports that investigated sinus anatomy used a descriptive, subjective method, i.e. the stenosis was diagnosed according to the impression of the radiologist (Bono et al., 2005; Dwyer et al., 2013; Farb et al., 2003; Horev et al., 2013). Such method is user dependent, based on the examiner's experience, and is of qualitative nature. The current study applies, for the first time, a method based on computer‐assisted cross‐sectional analysis. This enabled quantification of the TS stenosis and expression of its severity using exact cross‐sectional and volumetric data.

In contrast with other tools, the CAD method used in this study can be employed in cross‐sectional vessel analysis across different vascular systems in any desired plane, and is user independent. We validated the method by comparing to manual analysis, which was highly accordant (Farb et al., 2003).

In addition to the reported pathology of TS stenosis, our method added a new unreported feature. In IIH, we observed not only general narrowing of both transverse sinuses, but also the presence of a particularly narrowed segment. The precise detection of very thin vessels, with diameters up to 3 voxel units (1.5 mm), enabled us to identify this feature. Such abnormalities could not have been analyzed previously. While all investigated parameters demonstrated highly significant differences between IIH patients and controls, the largest difference was found when comparing the minimal cross‐sectional areas of TS. This is apparently due to this segment being highly stenotic in IIH patients.

Continuous CSF outflow seems to be enabled by the pressure gradient between the CSF and the venous sinuses. Any disturbance of this gradient may raise ICP. For example, in the case of sinus venous thrombosis: ICP increases secondary to increased sinus venous pressure, which enables continuous CSF outflow and filtration through the arachnoid granulations (Farb et al., 2003). Any stenotic morphology may be associated with a pressure gradient across the narrowed segment. Indeed, high intracranial venous sinus pressure has been shown in cases of IIH (Bono et al., 2005; Johnston et al., 2002). Still, the question as to whether stenosis in IIH is a cause of increased ICP or a sequel of it, is unclear. The reversibility of stenosis after ICP normalization supports the notion of a direct and dynamic relationship between ICP and the appearance of venous sinuses (Baryshnik & Farb, 2004; Horev et al., 2013; Rohr et al., 2012). This contrasts with the report of persistent transverse sinus stenosis after normalization of CSF pressure (Bono et al., 2005). An anatomic condition may contribute to the stenosis and explain those findings. Reduction in ICP by removal of CSF was shown to lower the venous sinus pressure (King, Mitchell, Thomson, & Tress, 1995). This led to the conclusion that increased venous pressure in IIH patients is caused by elevated ICP and not the reverse (Corbett & Digre, 2002). Recently accumulated evidence shows that dilation of the venous stenosis by stenting leads to immediate and long‐lasting relief of IIH symptoms (Donnet et al., 2008; Zheng, Zhou, Zhao, Zhou, & He, 2010). In any case, a positive feedback mechanism seems to be involved: venous hypertension proximal to the stenosis leads to further increased ICP, which then worsens the stenosis. Prospective cross‐sectional analysis of the TS before and after treatment using serial MRV studies over time may shed further light on this question, provide auxiliary information for follow up and contribute to decisions regarding stenting TS stenosis in IIH patients.

Computer assisted detection analysis of the TS also enabled evaluation of cross‐sectional vessel shape. A previous study by Farb, Forghani, Lee, Mikulis, and Agid (2007). reported a rather round shape for larger vessels in intracranial hypotension. However, this parameter was not previously investigated in IIH or in an objective quantitative manner. We found narrower vessels to have a more triangular shape than wider vessels, which were rounded. The pathophysiologic relationship between vessel shape and size has not been investigated. A possible explanation for our results is that the resistance of the TS vessel wall in IIH patients is greater than in controls and not homogenic. Though the vessel in the former is narrowed, it must withstand increased venous pressure. Consequently, the lumen tends to expand to the site of lower resistance and becomes more triangular. Further investigation of lumen resistance is needed to investigate this possibility.

We did not find a correlation between the degree of TS stenosis and opening pressure in LP. Farb et al. (2003) also described a lack of such correlation. This result may be biased by the retrospective design of our study. The interval between LP and the time of MRV was variable. The majority of patients underwent brain imaging more than one month after LP; still, in 10 of 38 patients (26%) less than one month passed between LP and MRV. However, we did not find outcome measures to change, when comparing the early and late groups. Further investigation with a fixed time interval between LP and imaging may reveal new findings. In any case, it would be preferable to perform the MRV prior to LP. Due to logistic necessities, this rarely occurs, as computed tomography scans are commonly performed on an urgent basis before LP. Another limitation of this study is that the examiner who ran the computerized analysis was unmasked to the group assignments of the subjects.

Although we did not find a correlation between BMI and severity of vessel thinning in the IIH group, we cannot reach a conclusion regarding the impact of BMI on dural sinuses in persons without IIH. Further studies in healthy individuals with high BMI may rule out a potential connection. As the aim of our study was to study TS configuration in IIH, patients with other conditions which raise ICP were not included. Further studies on other high pressure syndromes are needed in order to understand whether the narrowing is a specific feature associated with IIH or may be linked to high ICP itself. Should TS narrowing be unique to IIH, differential diagnosis could be eased using QIB. Furthermore, our control group included only subjects with normal brain MRI/MRV. We were able to identify specific anatomic patterns for healthy individuals.

In conclusion, we described an exact quantitative method, which can be easily applied to evaluate TS pathology. Our results showed significant differences between IIH patients and controls. CAD analysis appears as a precise tool for investigating mechanisms and responses to treatment in IIH patients.

## Conflict of Interest

The authors have no conflict of interest.
