# Supplementary material for: Quantitative imaging biomarkers for dural sinus patterns in idiopathic intracranial hypertension
Source: Brain Behav. 2017 Jan 3;7(2):e00613. doi: 10.1002/brb3.613 (PMC5318366; doi:10.1002/brb3.613)
Supplement: Supplementary file 1 [file BRB3-7-e00613-s001.docx]

**Table 1: Demographic data and baseline characteristics**

| Patient No. | Age | Gender | Opening pressure | Treatment | BMI | Time between LP and MRV |
| --- | --- | --- | --- | --- | --- | --- |
| 1 | 21 | F | 170 | 0 | 25.3 | <1Y |
| 2 | 22 | F | 410 | 0 | 23.6 | <1Y |
| 3 | 23 | F | 300 | MT | 29.3 | PRE |
| 4 | 57 | F | 212 | 0 | 29.2 | <1Y |
| 5 | 35 | F | 205 | MT | N/A | 1M |
| 6 | 18 | M | 310 | MT | 18.7 | 1M |
| 7 | 54 | F | 300 | 0 | 30.1 | <1Y |
| 8 | 30 | F | 240 | MT | N/A | 1M |
| 9 | 23 | F | 310 | MT | 27.2 | <1Y |
| 10 | 28 | M | 400 | MT | N/A | <1Y |
| 11 | 51 | F | 310 | VPS | N/A | PRE |
| 12 | 42 | F | 320 | 0 | 32.7 | >1Y |
| 13 | 25 | F | 290 | MT | 43.5 | <1Y |
| 14 | 34 | F | 260 | MT | 29.1 | 1M |
| 15 | 34 | F | 270 | MT | 29.0 | <1Y |
| 16 | 34 | F | 365 | 0 | 33.9 | PRE |
| 17 | 34 | F | 300 | MT | 31.5 | >1Y |
| 18 | 32 | F |  | MT | 39.1 | <1Y |
| 19 | 32 | F | 270 | MT | 30.8 | <1Y |
| 20 | 28 | F | 380 | MT | 42.2 | >1Y |
| 21 | 45 | F | 405 | 0 | 33.0 | <1Y |
| 22 | 27 | F | 380 | MT | 23.3 | <1Y |
| 23 | 34 | F | 370 | MT | 34.9 | <1Y |
| 24 | 21 | F | 370 | MT | 34.0 | <1Y |
| 25 | 26 | F | 300 | 0 | N/A | <1Y |
| 26 | 28 | F | 250 | MT | 28.4 | <1Y |
| 27 | 25 | F | 280 | 0 | 21.2 | <1Y |
| 28 | 34 | M | 290 | MT | 21.6 | >1Y |
| 29 | 29 | F | 420 | 0 | 48.3 | PRE |
| 30 | 58 | F | 370 | MT | N/A | >1Y |
| 31 | 31 | F | 550 | MT | 39.0 | 1M |
| 32 | 32 | F | 250 | MT | 37.0 | <1Y |
| 33 | 27 | F | 500 | MT | 41.9 | 1M |
| 34 | 36 | F | 250 | MT | 21.8 | >1Y |
| 35 | 32 | F | 250 | MT | 27.3 | <1Y |
| 36 | 28 | F | 420 | N/A | 44.0 | <1Y |
| 37 | 54 | F | 270 | MT | 28.3 | >1Y |
| 38 | 31 | M | 400 | MT | 43.0 | <1Y |

**MT=Medical treatment, VPS=Ventriculoperitoneal shunt**

**MRV before LP = pre**

**MRV <1month after LP = <1M**

**MRV 2-12 months after LP = <1Y**

**MRV >12 months after LP = >1Y**
