# Supplementary material for: Quantitative imaging biomarkers for dural sinus patterns in idiopathic intracranial hypertension
Source: Brain Behav. 2017 Jan 3;7(2):e00613. doi: 10.1002/brb3.613 (PMC5318366; doi:10.1002/brb3.613)
Supplement: Supplementary file 2 [file BRB3-7-e00613-s002.docx]

**Methods – Data analysis**

As an initial step, all images were registered and normalized using the SPM toolkit. An automatic vessel segmentation and extraction from the brain volume were performed using SPM brain masking, and a specially developed segmentation procedure based on region growing method. Volumetric skeletonization of the segmented vessel object was performed using the 3D Multistencil Fast Marching Method. Cross-sectional planes were determined at each skeleton point. As a final step of the algorithm a special clustering procedure was applied to define the most outer edge of a vessel object at each cross-sectional plane. To this end a connected vessel object with the centroid at a skeleton point was defined. Then, watershed transformation was employed to separate a single vessel cross-section from attached components like intersections with other vessels. Triangularity shape characteristics were used to quantify contour changes of the dural sinuses. To this end, the boundary points of vessel cross-sections were used to derive geometric measurements including circumference, area, and triangularity. The circumference, as polygon length (Lt), and the area (At) were calculated directly. Triangularity of the vessel cross-section was estimated as a ratio of the area of the biggest triangle inscribed into the polygon, to the area of the polygon. Circularity, which was a parameter of shape compactness, was defined as: 4πAt/Lt2.
